# Supplementary material for: The interactome of intact mitochondria by cross-linking mass spectrometry provides evidence for coexisting respiratory supercomplexes
Source: Mol Cell Proteomics. 2017 Dec 8;17(2):216–32. doi: 10.1074/mcp.RA117.000470 (PMC5795388; doi:10.1074/mcp.RA117.000470)
Supplement: Supplemental Data [file supp_RA117.000470_133922_0_supp_23978_fzffwf.zip › spectra_annotation/mito_spectra_annotation/408-1-1-1-6-1.pdf]

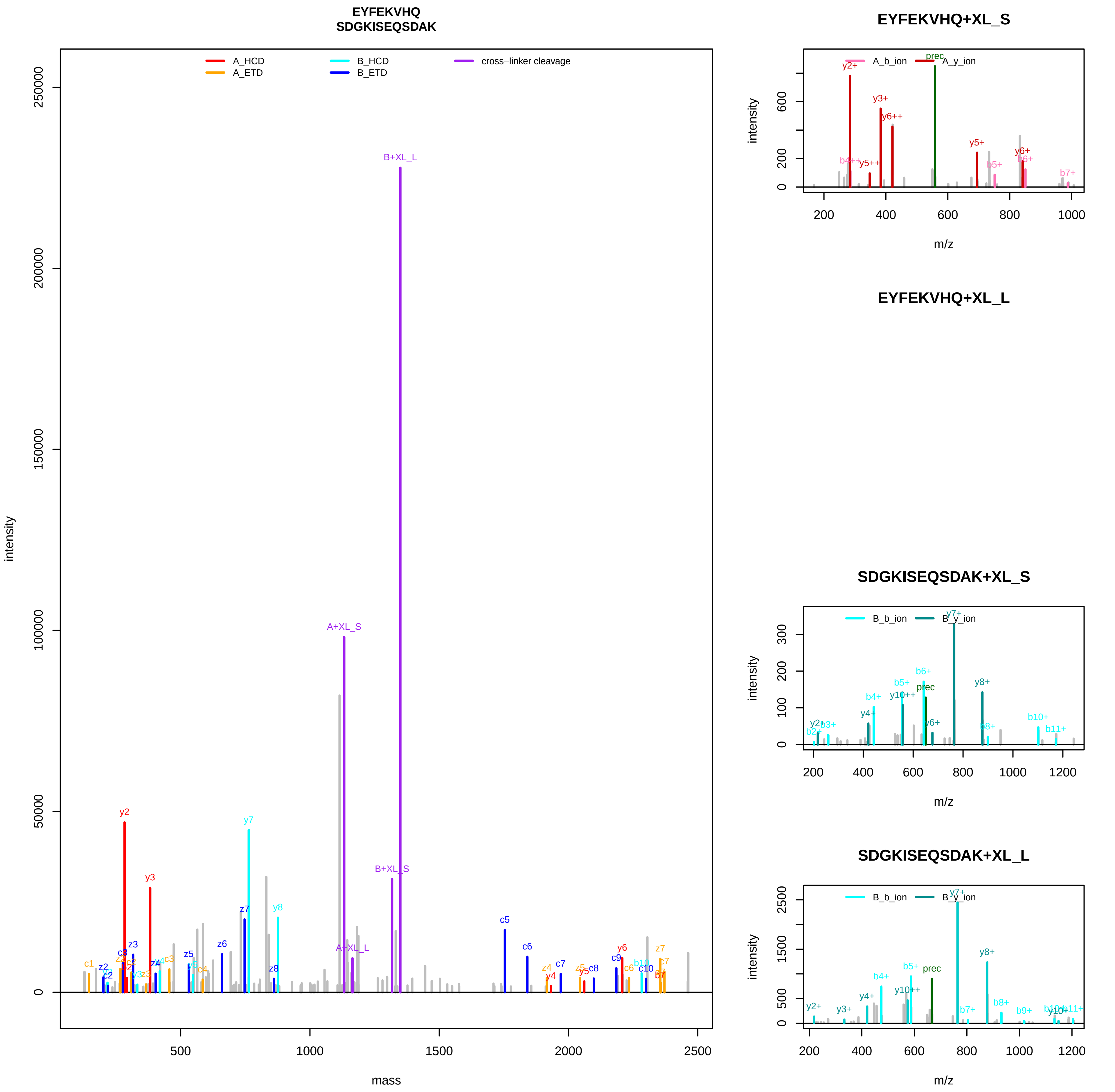

EYFEKVHQ
SDGKISEQSDAK
A_HCD
A_ETD
B_HCD
B_ETD
cross−linker cleavage
B+XL_L
A+XL_S
B+XL_S
A+XL_L
y2
y3
y6
y4
y5
y7
y8
c5
c6
c7
c8
c9
c10
z2
z3
z4
z5
z6
z7
z8
c1
c2
c3
c4
z4
z5
z7
b7
b10
c6
c7
intensity
mass
0
50000
100000
150000
200000
250000
500
1000
1500
2000
2500
EYFEKVHQ+XL_S
A_b_ion
A_y_ion
prec
y2+
y3+
y6++
y5+
y5++
y6+
b4++
b5+
b6+
b7+
intensity
m/z
0
200
600
200
400
600
800
1000
EYFEKVHQ+XL_L
SDGKISEQSDAK+XL_S
B_b_ion
B_y_ion
y7+
y8+
b6+
b5+
y10++
prec
b4+
y4+
y2+
b2+
b3+
y6+
b8+
b10+
b11+
intensity
m/z
0
100
200
300
200
400
600
800
1000
1200
SDGKISEQSDAK+XL_L
B_b_ion
B_y_ion
y7+
y8+
b5+
prec
b4+
y10++
y4+
y2+
y3+
b7+
b8+
b9+
b10+
y10+
b11+
intensity
m/z
0
500
1500
2500
200
400
600
800
1000
1200
